# Association between metabolic syndrome, macular structures and retinal vascular calibres in the Northern Finland Birth Cohort Eye Study

**DOI:** 10.1111/aos.70131

**Published:** 2026-04-17

**Authors:** Jenni M. Huru, Oona Ahokas, Ilmari L. Leiviskä, Ville O. Saarela, M. Johanna Liinamaa

**Affiliations:** ^1^ Department of Ophthalmology Oulu University Hospital Oulu Finland; ^2^ MRC Oulu Oulu Finland; ^3^ Research Unit of Clinical Medicine University of Oulu Oulu Finland; ^4^ Department of Mathematical Sciences University of Oulu Oulu Finland

**Keywords:** clinical trial, epidemiology, macula, metabolic syndrome, retina

## Abstract

**Purpose:**

We aimed to evaluate the association of metabolic syndrome (MetS) with macular thickness and retinal vascular calibres in the population‐based cohort (Northern Finland Birth Cohort).

**Methods:**

The population of 2242 individuals was divided into MetS (*n* = 344) and control groups (*n* = 1898). Cirrus HD‐OCT 4000 was used to measure total macular thickness. Central retinal arteriolar equivalent (CRAE) and central retinal venular equivalent (CRVE) were calculated from the fundus images. The MetS was defined according to the National Cholesterol Education Program (Adult Treatment Panel III).

**Results:**

A significant reduction in macular thickness was observed in subjects with MetS (279.4 ± 13.5 vs. 282.2 ± 14.1 μm, *p* < 0.001); the difference was greatest in the nasal and inferior subfields in the outer perimeter (−3.18 μm, *p* < 0.001 and − 3.68 μm, *p* < 0.001). Among the MetS components, waist circumference (WC) was particularly associated with thinning of the macula. Furthermore, subjects with MetS displayed decreased CRAE (139.8 ± 13.2 vs. 141.8 ± 14.0 μm, *p* = 0.014) and increased CRVE (221.7 ± 20.5 vs. 218.3 ± 19.0 μm, *p* = 0.007). The elevated systolic blood pressure and WC were associated with arterial narrowing, while dyslipidemia, hypertriglyceridemia and low HDL cholesterol were associated with venular widening.

**Conclusion:**

We detected a thinning of the macula in the MetS in our population‐based cohort. The diameters of the retinal arteries decreased, and the venules increased in MetS. These findings suggest that obesity is related to structural changes in the eyes in middle‐aged subjects, which may have implications for ocular health.

## 1 INTRODUCTION

Metabolic syndrome (MetS) is defined as a combination of metabolic dysfunctions that increase the risk factors for cardiovascular diseases (CVDs) and type 2 diabetes mellitus (T2DM) (Alberti et al., 2009). National Cholesterol Education Program Adult Treatment Panel III (NCEP ATP III) has defined MetS as three or more of the following: increased waist circumference (WC), elevated triglycerides, low high‐density lipoprotein (HDL) cholesterol, elevated blood pressure and impaired glucose metabolism (NCEP, 2001).

The pathogenesis of MetS is not yet fully understood, but abdominal obesity and insulin resistance are considered key contributors (Alberti et al., 2009). Abdominal fat indirectly interferes with multiple physiological pathways, including glucose regulation, lipid metabolism and blood pressure control, and contributes to the development of MetS (Srikanthan et al., 2016). We previously demonstrated that impaired glucose metabolism, a component of MetS, is associated with macular thinning (Huru et al., 2021), and Weisner et al. (2021) reported that retinal structural and metabolic abnormalities are associated with disease activity in diabetic retinopathy. Obesity (Laiginhas et al., 2019) and MetS (Karaca & Karaca, 2018; Zarei et al., 2017) have also been linked to thinning of the peri‐papillary nerve fibre layer (RNFL), reflecting neurodegenerative changes. Furthermore, a reduction in photoreceptor layer thickness has been observed in patients with MetS (Karaca & Karaca, 2018).

Although MetS has been considered a large‐vessel disease, growing evidence indicates that it also affects the microvasculature in a manner comparable to diabetes (Wong et al., 2004). Previous studies have established an association between MetS and wider retinal venules (Kawasaki et al., 2008; Wong et al., 2004) and narrower retinal arterioles (Wang et al., 2016; Wong et al., 2004; Yuan et al., 2012).

This study aimed to evaluate the thickness of the macula and the calibres of the retinal arteries and venules in subjects with MetS in a large population‐based birth cohort.

## 2 METHODS

### 2.1 Study population

The Northern Finland Birth Cohort (NFBC) 1966 is a population‐based birth cohort covering 12 058 subjects whose expected birthdate was in 1966. The NFBC represents a homogenous population in terms of age, ethnicity and living environment. A follow‐up study of 46‐year‐olds was performed from 2012 to 2015, and various clinical examinations were performed, including cardiorespiratory, physiological, orthopaedic, cognitive, dermatological, ophthalmological and extensive laboratory tests. The number of participating subjects in the NFBC eye screening group were 3070, and the study population with no missing data was 2242, including 344 subjects with MetS. In our analyses, we have used data from the right eye of each participant. The NFBC Eye Study design has previously been described (Saarela et al., 2013), approved by the Ethical Committee of the Northern Ostrobothnia Hospital District (no. 94/2011), and followed the principles of the Declaration of Helsinki.

### 2.2 Outcome variables

#### 2.2.1 The optical coherence tomography (OCT)

The OCT was obtained using the Cirrus HD‐OCT 4000 imagine device (software version 6.0.0; Carl Zeiss Meditec) with a macular cube 512 × 128 scan protocol. This protocol forms a 6 × 6 × 2 mm^3^ cube and constructs a profile of the total macular volume, average macular thickness and average thicknesses of the nine macular subfields. The macular cube protocol includes a ganglion cell (GC) protocol, which measures the thickness of the sum of the ganglion cell layer (GCL) and inner plexiform layer (IPL) in six macular subfields (superior, nasal superior, temporal superior, inferior, nasal inferior, temporal inferior). These two layers and the retinal nerve fibre layer (RNFL) form a ganglion cell complex (GCC).

#### 2.2.2 Fundus images

As presented earlier (Geneid et al., 2019), retinal arteriolar and venular calibres were measured from the fundus images. These images were photographed with a canon CF‐60DSi digital Mydriatic fundus camera with an attached canon EOS‐1Ds MK III SLR digital camera (Canon Inc., Tokyo, Japan) (Saarela et al., 2013). A single grader using a semiautomatic computer‐assisted integrative vessel analysis software (IVAN, University of Wisconsin, Madison, WI, USA) analysed the digital fundus images of the right eye (Knudtson et al., 2003). This software detects the optic disc and divides the image into three concentric zones. After identifying the optic disc and zones, the six biggest arterioles and venules were tracked, and the IVAN software calculated retinal vessel calibres. Three summary variables were created: Central retinal arteriolar equivalent (CRAE), central retinal venular equivalent (CRVE) and arteriovenous ratio (AVR) as the ratio of the two previous variables (Knudtson et al., 2003). There were three graders all blinded to the participant's characteristics and the intra‐grader variability was 4.5% for CRAE and 3.7% for CRVE; the inter‐grader variability was 3.7% for CRAE and 3.0% for CRVE (Geneid et al., 2019).

#### 2.2.3 MetS

The MetS was defined according to the National Cholesterol Education Program (Adult Treatment Panel III or ATP III). Subjects with three or more of the following criteria were classified as having MetS: WC >102 cm in men and >88 cm in women, triglycerides ≥1.7 mmol/L, HDL cholesterol <1.0 mmol/L in men and <1.3 mmol/L in women, fasting plasma glucose ≥6.1 mmol/L and systolic blood pressure ≥130 mmHg or diastolic ≥85 mmHg (NCEP, 2001). The ATP III criteria were chosen because of their widespread use in epidemiological research and to ensure comparability with previous studies.

WC was measured from the middle between the lowest rib and the iliac crest. The plasma glucose and lipids were calculated from the blood sample after at least 10 h of overnight fasting. Systolic and diastolic blood pressure were measured after 15 min of rest with an automatic blood pressure monitor (Omron M10‐IT, Omron Healthcare, Kyoto, Japan), and the minimum value of three measurements was used (Nordström et al., 2021).

### 2.3 Statistical analysis

The equalities between the group means were tested with Welch's *T*‐test, which does not assume equal variances or sample sizes for the groups. When data was not of normal distribution, the Mann–Whitney *U* test tested the equality of distributions. We used multivariate regression models, adjusted for sex and spherical equivalent, to assess the relationship between MetS and macular thickness. All the numerical results were calculated using IBM SPSS Statistics version 29.0 and graphical line art with R program version 4.0.0. A *p*‐value of 0.05 or lower was considered statistically significant except for the macular thickness map where we applied Bonferroni correction and the corrected level of significance was *p* < 0.005.

## 3 RESULTS

### 3.1 Baseline characteristics

All participants were born between 1965 and 1967; the mean age at the time of the data collection was 47.5. Of the 2242 participants, 344 met the criteria for MetS. Table 1 lists the baseline characteristics of MetS and the control group. Components of the MetS in the two groups were as follows: median WC 107 cm (102–115) in the MetS group and 88 cm (80–96) (*p* < 0.001) in the control group, triglycerides 1.91 mmol/L (1.45–2.50) and 0.96 mmol/L (0.73–1.27) (*p* < 0.001), HDL cholesterol 1.2 ± 0.3 and 1.6 ± 0.4 mmol/L (*p* < 0.001), fasting plasma glucose 6.3 ± 1.6 and 5.4 ± 0.5 mmol/L (*p* < 0.001), systolic blood pressure 132.4 ± 13.5 and 118.6 ± 14.4 mmHg (*p* < 0.001) and diastolic blood pressure 91.5 ± 9.1 and 80.4 ± 9.6 mmHg (*p* < 0.001), respectively.

**TABLE 1:** Baseline characteristics of the study population (*N* = 2242).

	MetS (*N* = 344)	Controls (*N* = 1898)	*p*
Age (years)	47.4 (46.6 to 48.0)	47.5 (46.5 to 48.0)	0.903
Male (%)	184 (53%)	784 (41%)	**<0.001**
BMI (kg/m^2^)	31.5 (29.1 to 35.0)	25.1 (23.0 to 27.9)	**<0.001**
Waist circumference (cm)	107 (102 to 115)	88 (80 to 96)	**<0.001**
Fasting plasma glucose (mmol/L)	6.3 (1.6)	5.4 (0.5)	**<0.001**
HbA1C (mmol/L)	41.2 (10.3)	35.9 (4.4)	**<0.001**
Diastolic blood pressure (mmHg)	91.5 (9.1)	80.4 (9.6)	**<0.001**
Systolic blood pressure (mmHg)	132.4 (13.5)	118.6 (14.4)	**<0.001**
HDL cholesterol (mmol/L)	1.2 (0.3)	1.6 (0.4)	**<0.001**
LDL cholesterol (mmol/L)	3.8 (1.0)	3.4 (0.9)	**<0.001**
Total cholesterol (mmol/L)	5.6 (1.0)	5.3 (0.9)	**<0.001**
Triglycerides (mmol/L)	1.91 (1.45 to 2.50)	0.96 (0.73 to 1.27)	**<0.001**
Intra‐ocular pressure (mmHg)	15.9 (3.6)	14.6 (3.3)	**<0.001**
Spherical equivalent	−0.38 (−2.13 to 0.25)	−0.38 (−2.0 to 0.38)	0.274
Central retinal arteriolar equivalent (μm)	139.8 (13.2)	141.8 (14.0)	**0.014**
Central retinal venular equivalent (μm)	221.7 (20.5)	218.3 (19.0)	**0.007**
Macula cube volume (mm^3^)	10.0 (0.5)	10.1 (0.5)	**0.001**
Macula cube thickness (μm)			
Average (μm)	279.4 (13.5)	282.2 (14.1)	**<0.001**
Central subfield thickness (μm)	260.3 (20.1)	260.8 (21.2)	0.688
Inner nasal macular thickness (μm)	325.8 (16.7)	327.4 (17.2)	0.108
Inner superior macular thickness (μm)	322.6 (16.2)	325.0 (16.7)	**0.012**
Inner temporal macular thickness (μm)	311.6 (16.1)	313.4 (16.2)	0.051
Inner inferior macular thickness (μm)	319.5 (17.1)	322.0 (17.1)	**0.012**
Outer nasal macular thickness (μm)	294.2 (15.6)	297.1 (16.3)	**0.001**
Outer superior macular thickness (μm)	277.6 (14.2)	280.4 (14.7)	**0.001**
Outer temporal macular thickness (μm)	260.8 (13.4)	262.9 (14.2)	**0.005**
Outer inferior macular thickness (μm)	266.7 (13.9)	270.2 (15.3)	**<0.001**

*Note*: Normally distributed variables are presented as mean and standard deviation; skewed variables are presented as median and inter‐quartile range (from first to third quartile). Significant *p* values are shown in bold.

### 3.2 Macular thickness

As shown in Table 1, the average thickness of the macula was lower in participants with MetS compared with controls (279.4 ± 13.5 vs. 282.2 ± 14.1 μm, *p* < 0.001). Central subfield thickness did not differ between the groups (*p* = 0.688). However, decreased thickness was observed in several other macular cube subfields. In the inner macular ring, the MetS group exhibited reduced thickness in the superior (322.6 ± 16.2 vs. 325.0 ± 16.7 μm, *p* = 0.012) and inferior sectors (319.5 ± 17.1 vs. 322.0 ± 17.1 μm, *p* = 0.012), while nasal and temporal sectors showed no significant difference. In the outer macular ring, all four sectors were thinner in the MetS group: nasal (294.2 ± 15.6 vs. 297.1 ± 16.3 μm, *p* = 0.001), superior (277.6 ± 14.2 vs. 280.4 ± 14.7 μm, *p* = 0.001), temporal (260.8 ± 13.4 vs. 262.9 ± 14.2 μm, *p* = 0.005) and inferior (266.7 ± 13.9 vs. 270.2 ± 15.3 μm, *p* < 0.001).

Linear regression analyses confirmed that MetS was associated with reduced macular thickness across several subfields (Table 2). After adjusting for sex and spherical equivalent, the average macular thickness was significantly lower in participants with MetS (*b* = −3.00, *p* < 0.001). Significant reductions were observed in all macular subfields except the central and inner nasal, with the largest effects in the inner inferior (*b* = −3.45, *p* < 0.001) and outer inferior (*b* = −3.68, *p* < 0.001) regions. All models were adjusted for sex and spherical equivalent.

**TABLE 2:** Linear regression analysis for retinal vascular and macular parameters with metabolic syndrome as a predictor, adjusted for sex and spherical equivalent (*n* = 2242).

	*b*	*p*	Lower	Upper
Central retinal arteriolar equivalent (μm)	−2.03	**0.016**	−3.69	−0.37
Central retinal venular equivalent (μm)	3.23	**0.006**	0.95	5.52
Macula cube average thickness (μm)	−3.00	**<0.001**	−4.56	−1.44
Central subfield thickness (μm)	−2.08	0.076	−4.37	0.22
Inner nasal macular thickness (μm)	−2.59	0.007	−4.48	−0.70
Inner superior macular thickness (μm)	−3.08	**0.001**	−4.94	−1.22
Inner temporal macular thickness (μm)	−2.80	**0.002**	−4.57	−1.03
Inner inferior macular thickness (μm)	−3.45	**<0.001**	−5.35	−1.56
Outer nasal macular thickness (μm)	−3.18	**<0.001**	−5.04	−1.33
Outer superior macular thickness (μm)	−2.56	**0.002**	−4.20	−0.91
Outer temporal macular thickness (μm)	−2.52	**0.001**	−4.04	−1.00
Outer inferior macular thickness (μm)	−3.68	**<0.001**	−5.39	−1.97

*Note*: Significant *p* values are shown in bold.

Figure 1 visualises the differences in macular thickness in each subfield between the MetS and control groups, adjusted for spherical equivalent and sex. Overall, the MetS group showed reduced macular thickness in all subfields except the central and inner nasal. We also performed additional analyses of the GCL and RNFL thicknesses (data not shown), but no significant differences were observed between participants with MetS and controls.

**FIGURE 1:**
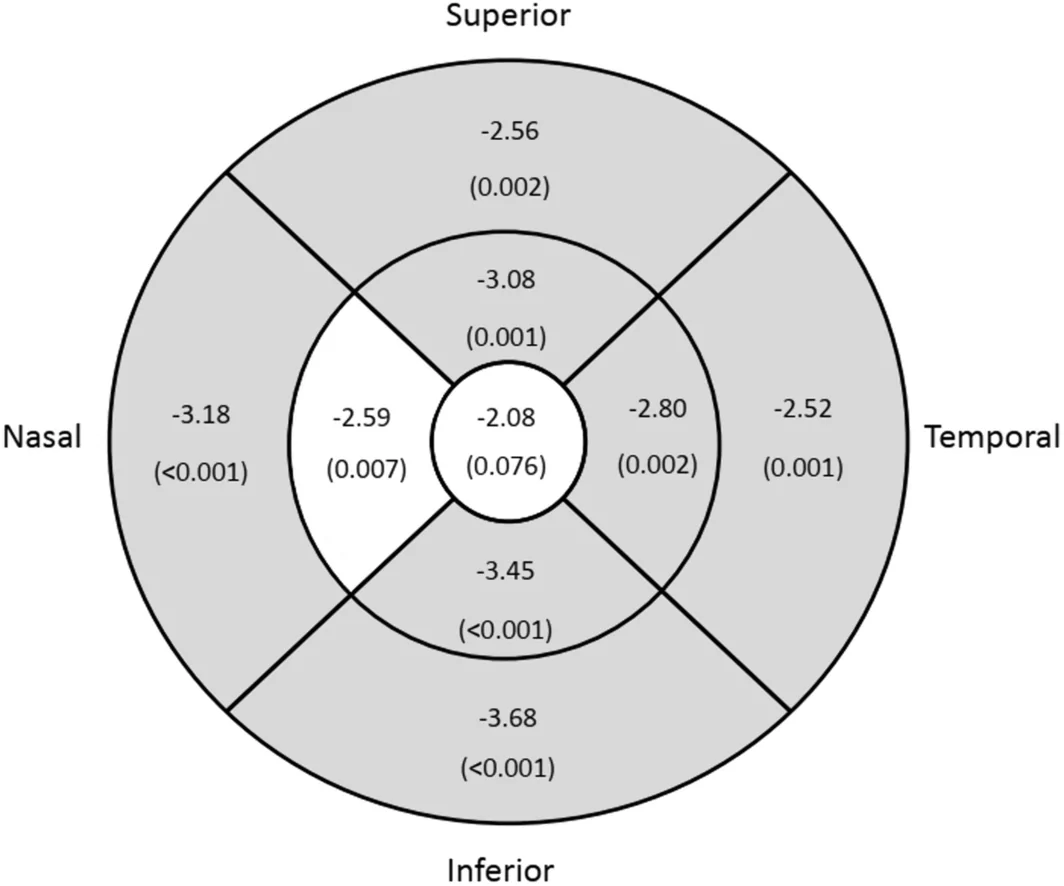
Mean differences (μm) in macular subfield thicknesses between MetS group (*n* = 344) and the control group (*n* = 1898), adjusted for sex and spherical equivalent. Subfields remaining significant after Bonferroni correction are shaded in grey; *p*‐values are shown in parentheses.

To examine the association between macular thickness and individual components of MetS, we compared group means of macular subfield thickness within each component of MetS (Table 3). Our data indicate that increased WC (>102 cm in men, >88 cm in women) was associated with reduced macular thickness in all subfields except central. Other MetS components showed more localised effects: elevated blood pressure (≥130/85 mmHg) was associated with increased central subfield thickness (*p* = 0.017), elevated fasting plasma glucose (≥6.1 mmol/L) was associated with decreased outer superior subfield thickness (*p* = 0.012), and high triglycerides (≥1.7 mmol/L) were associated with reduced thickness in the outer superior and inferior subfields.

**TABLE 3:** The macula's thickness and components of the MetS (*N* = 2242).

Macula	Inner										Outer							
	Central subfield		Nasal		Superior		Temporal		Inferior		Nasal		Superior		Temporal		Inferior	
	Mean (μm)	SD	Mean (μm)	SD	Mean (μm)	SD	Mean (μm)	SD	Mean (μm)	SD	Mean (μm)	SD	Mean (μm)	SD	Mean (μm)	SD	Mean (μm)	SD
Overall	260.7	21.1	327.1	17.1	324.6	16.7	313.1	16.2	321.6	17.1	296.7	16.3	279.9	14.6	262.6	14.1	269.7	15.2
Waist circumference >102 cm in men; >88 cm in women																		
No (*N* = 1430)	261.3	21.7	**327.7**	**17.4**	**325.3**	**16.6**	**313.8**	**16.3**	**322.3**	**17.5**	**297.3**	**16.2**	**280.4**	**14.4**	**263.2**	**14.3**	**270.3**	**15.4**
Yes (*N* = 812)	259.8	19.9	**326.2**	**16.6**	**323.4**	**16.8**	**312.1**	**16.0**	**320.3**	**16.5**	**295.7**	**16.3**	**279.0**	**15.0**	**261.6**	**13.9**	**268.6**	**14.6**
*p*	0.098		**0.044**		**0.009**		**0.017**		**0.008**		**0.025**		**0.026**		**0.011**		**0.010**	
Fasting plasma glucose ≥6.1 mmol/L																		
No (*N* = 1956)	260.5	20.9	327.2	17.1	324.8	16.7	313.3	16.2	321.8	17.0	296.9	16.3	**280.2**	**14.6**	262.7	14.2	269.9	15.2
Yes (*N* = 286)	262.4	22.3	326.9	17.5	323.5	16.9	312.3	16.3	320.4	18.2	295.2	15.6	**277.9**	**14.6**	262.1	13.8	268.2	15.1
*p*	0.179		0.807		0.236		0.372		0.223		0.086		**0.012**		0.528		0.085	
Blood pressure ≥130/85 mmHg																		
No (*N* = 1298)	**259.8**	**21.8**	326.8	17.1	324.6	16.4	313.0	16.0	321.6	16.8	296.9	16.0	280.3	14.4	262.8	14.0	270.2	14.8
Yes (*N* = 944)	**261.9**	**20.0**	327.6	17.2	324.7	17.1	313.4	16.4	321.6	17.6	296.4	16.6	279.4	15.0	262.4	14.4	269.0	15.7
*p*	**0.017**		0.307		0.942		0.574		0.951		0.512		0.167		0.525		0.060	
Triglycerides ≥1.7 mmol/L																		
No (*N* = 1841)	260.4	21.0	327.1	17.1	324.7	16.7	313.1	16.1	321.7	17.1	297.0	16.4	**280.2**	**14.6**	262.7	14.2	**270.0**	**15.4**
Yes (*N* = 401)	262.0	21.4	327.3	17.1	324.3	16.8	313.3	16.5	321.3	17.5	295.4	15.8	**278.4**	**14.9**	262.2	13.9	**268.3**	**14.0**
*p*	0.171		0.885		0.680		0.823		0.678		0.083		**0.030**		0.480		**0.034**	
HDL <1.0 mmol/L in men; <1.3 mmol/L in women																		
No (*N* = 1973)	261.0	21.0	327.3	17.2	324.8	16.8	313.3	16.3	321.8	17.2	296.8	16.5	279.9	14.8	262.8	14.3	269.8	15.3
Yes (*N* = 269)	258.5	21.5	325.7	16.1	323.5	15.6	311.7	15.5	319.9	16.6	295.7	14.7	279.6	13.5	261.3	13.1	269.1	14.0
*p*	0.067		0.110		0.196		0.112		0.083		0.246		0.693		0.076		0.463	

*Note*: Statistically significant values are highlighted.

In sex‐stratified analyses, the associations between MetS components and macular thickness showed some sex‐specific patterns. Detailed results for the inner and outer macular subfields in men and women are presented in the Tables S1 and S2. Among men, increased WC was associated with thinner macular thickness across multiple inner macular subfields. In addition, elevated fasting plasma glucose, blood pressure and triglyceride levels were associated with reduced thickness in selected macular subfields (Table S1). In women, several components of the MetS were also associated with reduced macular thickness, with particularly pronounced associations observed for elevated fasting plasma glucose and triglyceride levels (Table S2).

### 3.3 Retinal vessel calibres CRAE and CRVE

Table 1 shows that arterial diameters were decreased, and venular diameters were increased in participants with MetS. CRAE was 139.8 ± 13.2 μm in the MetS group versus 141.8 ± 14.0 μm in controls (*p* = 0.014), while CRVE was 221.7 ± 20.5 versus 218.3 ± 19.0 μm (*p* = 0.007). Linear regression analyses (Table 2) confirmed these findings, with MetS being associated with narrower CRAE (*b* = −2.03, *p* = 0.016) and wider CRVE (*b* = 3.23, *p* = 0.006).

To evaluate the association between retinal vessel calibres and individual MetS components, we used Welch's *t*‐test to compare the mean levels of CRAE and CRVE across the presence or absence of each component (Table 4). CRAE was lower in participants with increased WC (139.7 ± 14.0 vs. 142.5 ± 13.8 μm, *p* < 0.001) and in those with elevated blood pressure (137.2 ± 13.0 vs. 144.5 ± 13.8 μm, *p* < 0.001). No significant differences in CRAE were observed for the remaining MetS components. CRVE did not differ significantly by WC or blood pressure but was higher in participants with elevated fasting plasma glucose (221.8 ± 19.6 vs. 218.3 ± 19.2 μm, *p* = 0.008), elevated triglycerides (221.0 ± 20.5 vs. 218.3 ± 19.0 μm, *p* = 0.024) and low HDL cholesterol (222.1 ± 21.1 vs. 218.3 ± 19.0 μm, *p* = 0.009).

**TABLE 4:** Comparison of retinal arteriolar (CRAE) and venular (CRVE) diameters in participants with and without individual metabolic syndrome components.

	CRAE		CRVE	
	Mean (μm)	SD	Mean (μm)	SD
Overall	141.5	13.9	218.8	19.3
Waist circumference >102 cm in men; >88 cm in women				
No (*N* = 1299)	142.5	13.8	218.5	19.2
Yes (*N* = 746)	139.7	14.0	219.3	19.5
*p*	**<0.001**		0.334	
Fasting plasma glucose ≥6.1 mmol/L				
No (*N* = 1790)	141.4	13.9	218.3	19.2
Yes (*N* = 255)	141.7	13.8	221.8	19.6
*p*	0.808		**0.008**	
Blood pressure ≥130/85 mmHg				
No (*N* = 1195)	144.5	13.8	219.0	19.1
Yes (*N* = 850)	137.2	13.0	218.5	19.6
*p*	**<0.001**		0.577	
Triglycerides ≥1.7 mmol/L				
No (*N* = 1692)	141.6	13.9	218.3	19.0
Yes (*N* = 353)	140.7	14.1	221.0	20.5
*p*	0.282		**0.024**	
HDL <1.0 mmol/L in men; <1.3 mmol/L in women				
No (*N* = 1801)	141.3	13.9	218.3	19.0
Yes (*N* = 244)	142.5	13.9	222.1	21.1
*p*	0.229		**0.009**	

*Note*: Significant *p* values are shown in bold.

## 4 DISCUSSION

This study aimed to evaluate macular thickness and retinal arteriolar and venular calibres in individuals with MetS compared with healthy controls in a large population‐based cohort. The main findings were as follows:

1. MetS was associated with a systematic pattern of macular thinning, with WC being the component of MetS most strongly associated with macular thickness.
2. Participants with MetS displayed decreased CRAE and increased CRVE. Elevated systolic blood pressure showed the strongest association with arteriolar narrowing, while low HDL cholesterol was most strongly associated with venular widening.

Thinning of the macular layers is proposed to reflect early neurodegenerative changes in several neurological diseases (Chan et al., 2019; Frohman et al., 2008; Wu et al., 2020). Thinning of the RNFL indicates the loss of GC axons, whereas loss of GC neurons contributes more broadly to thinning of the entire macula. Previously, a thicker macula has been associated with better visual acuity and self‐reported visual functions (Poh et al., 2020) whereas a thinner GCL is linked to visual impairment (Garzone et al., 2023) and together with thinner RNFL to reduced contrast sensitivity (Dong et al., 2022). Furthermore, thinning of different retinal layers has been associated with loss of total brain volume, grey and white matter and hippocampus volumes (Garzone et al., 2023; Mauschitz et al., 2022; Mutlu et al., 2017).

Only a few previous studies have examined the retina using OCT in MetS, mostly focusing on RNFL measurements. Some studies have reported a loss of peri‐papillary RNFL thickness in obesity (Laiginhas et al., 2019) and others in MetS (Karaca & Karaca, 2018; Zarei et al., 2017). However, macular changes in MetS remain poorly understood. Since the macular area contains over 50% of all GC bodies, and GC bodies are much larger than their axons, macular thinning may be a more sensitive marker of neurodegeneration than RNFL (Chan et al., 2019). Our study found reduced macular thickness in participants with MetS compared with healthy controls. These findings are consistent with those of Karaca and Karaca (2018) and Polat et al. (2023), who reported a reduction in the central macular thickness in MetS, without differences in the peri‐papillary or macular RNFL.

A strong association has been reported between obesity and Alzheimer's disease and other forms of dementia (Pedditizi et al., 2016; Qu et al., 2020), as well as between central obesity and grey matter changes in the brain (Pflanz et al., 2022), suggesting a potential link between obesity and retinal neurodegeneration. Our findings support this theory: in our study, WC was the component of MetS most strongly associated with macular thinning. To our knowledge, no previous study has reported a direct association between WC and macular thinning.

Our data indicate that subtle retinal vascular changes occur in subjects with MetS even before clinically detectable signs of retinal vasculopathy. These findings are largely consistent with previous reports. Specifically, we observed decreased retinal arterial diameters and increased venular diameters in subjects with MetS. Similar patterns of arterial narrowing have been reported in the Handan Eye Study (Zhao et al., 2012), the Atherosclerosis Risk in Communities Study (Wong et al., 2004) and the Australian Heart Eye Study (Wang et al., 2016). Increased retinal venular diameters have also been associated with MetS in several studies (Kawasaki et al., 2008; Saito et al., 2011; Wong et al., 2004), although Zhao et al. (2012) did not find a significant association. Furthermore, Japanese studies reported that both arterial narrowing and increased venular diameters were associated with a higher prevalence of MetS (Saito et al., 2011, 2015).

CVD remains one of the leading causes of death worldwide, and evaluating its risk factors has implications for individual morbidity and mortality as well as for healthcare costs. MetS represents the co‐occurrence of several CVD risk factors; therefore, examining its effects on the retina is clinically relevant, as retinal vessels and layers are the only areas of the microvasculature and neural system that can be easily and non‐invasively inspected and measured. Our findings suggest that abdominal obesity may be associated with structural changes in the retina that could compromise vision and ocular health over time. Weight control may help prevent retinal neurodegeneration and microvascular changes, at least to some extent. These conclusions are supported by an Italian study in which 29 severely obese patients underwent bariatric surgery: weight reduction and improvement in MetS components were associated with increased RNFL and foveal thickness (Posarelli et al., 2019).

This study's major strength lies in its NFBC Eye Study population, which is homogenous in terms of age, living environment and Caucasian ethnicity. Standardised assessments were performed for WC, cholesterol levels, blood pressure, glucose metabolism, OCT and fundus imaging. The main limitation is its cross‐sectional design, limiting further assessment of causality. Furthermore, the analysis is limited to middle‐aged Caucasian participants.

In summary, in this population‐based cohort, we detected a significant macular thinning in the MetS group, with WC being the most influential component. Retinal arterial diameters were decreased, whereas venular diameters were increased in participants with MetS. To our knowledge, this study is the first to demonstrate an association between obesity and subtle retinal structural changes in middle‐aged individuals, which may have implications for ocular health.

## Supporting information


Table S1.

Table S2.
