# Supplementary material for: Association between metabolic syndrome, macular structures and retinal vascular calibres in the Northern Finland Birth Cohort Eye Study
Source: Acta Ophthalmol. 2026 Apr 17;104(7):760–8. doi: 10.1111/aos.70131 (PMC13651912; doi:10.1111/aos.70131)
Supplement: Supplementary file 1 [file AOS-104-760-s001.docx]

Supplementary table 2. The macula’s thickness and the components of the MetS (N=2242) for women. Statistically significant values are highlighted.

| **FEMALES** | **Inner** | | | | | | | | | | **Outer** | | | | | | | |
| --- | --- | --- | --- | --- | --- | --- | --- | --- | --- | --- | --- | --- | --- | --- | --- | --- | --- | --- |
| **Macula** | Central subfield | | Nasal | | Superior | | Temporal | | Inferior | | Nasal | | Superior | | Temporal | | Inferior | |
|  | Mean (μm) | SD | Mean (μm) | SD | Mean (μm) | SD | Mean (μm) | SD | Mean (μm) | SD | Mean (μm) | SD | Mean (μm) | SD | Mean (μm) | SD | Mean (μm) | SD |
| **Overall** | 255.0 | 20.0 | 323.1 | 16.2 | 321.3 | 16.0 | 309.0 | 15.3 | 317.8 | 16.4 | 295.5 | 16.1 | 279.4 | 14.8 | 260.1 | 14.0 | 268.9 | 15.3 |
| **Waist circumference** >88cm |  | | | | | | | | | | | | | | | | | |
| No (N=771) | 254.3 | 20.7 | 322.7 | 16.3 | 321.6 | 15.8 | 309.0 | 15.2 | 318.0 | 16.8 | 295.8 | 15.9 | 279.9 | 14.5 | 260.5 | 14.1 | 269.5 | 15.6 |
| Yes (N=505) | 256.1 | 19.4 | 323.6 | 16.1 | 321.0 | 16.3 | 309.1 | 15.5 | 317.6 | 15.9 | 295.0 | 16.3 | 278.6 | 15.3 | 259.5 | 13.9 | 268.1 | 14.8 |
| P | 0.124 | | 0.365 | | 0.559 | | 0.931 | | 0.642 | | 0.376 | | 0.138 | | 0.225 | | 0.108 | |
| **Fasting plasma glucose** ≥6.1mmol/l |  | | | | | | | | | | | | | | | | | |
| No (N=1177) | 255.3 | 20.2 | 323.3 | 16.1 | **321.7** | **15.9** | **309.4** | **15.3** | **318.2** | **16.2** | 295.7 | 16.1 | **279.7** | **14.7** | 260.3 | 14.1 | 269.1 | 15.3 |
| Yes (N=99) | 251.1 | 20.4 | 319.9 | 16.7 | **317.7** | **16.3** | **305.0** | **14.6** | **313.4** | **18.4** | 293.5 | 15.7 | **275.6** | **16.4** | 257.8 | 13.2 | 266.2 | 14.9 |
| P | 0.050 | | 0.051 | | **0.021** | | **0.005** | | **0.013** | | 0.190 | | **0.018** | | 0.072 | | 0.066 | |
| **Blood pressure** ≥130/85mmHg |  | | | | | | | | | | | | | | | | | |
| No (N=851) | 255.0 | 20.7 | 323.2 | 16.2 | 321.8 | 15.8 | 309.3 | 15.2 | 318.3 | 16.2 | 295.6 | 15.9 | 279.7 | 14.6 | 260.5 | 13.8 | 269.5 | 14.9 |
| Yes (N=425) | 255.0 | 19.1 | 322.8 | 16.2 | 320.5 | 16.2 | 308.5 | 15.5 | 316.8 | 16.8 | 295.2 | 16.4 | 278.7 | 15.2 | 259.3 | 14.5 | 267.8 | 16.1 |
| P | 0.965 | | 0.709 | | 0.209 | | 0.358 | | 0.137 | | 0.675 | | 0.263 | | 0.145 | | 0.079 | |
| **Triglycerides** ≥1.7mmol/l |  | | | | | | | | | | | | | | | | | |
| No (N=1138) | 255.3 | 20.2 | 323.3 | 16.1 | **321.7** | **15.8** | **309.4** | **15.2** | **318.3** | **16.2** | 295.8 | 15.9 | **279.7** | **14.4** | **260.5** | **13.9** | **269.3** | **15.3** |
| Yes (N=138) | 252.9 | 20.4 | 321.0 | 17.2 | **318.2** | **17.0** | **306.3** | **15.8** | **314.1** | **17.9** | 293.3 | 17.2 | **276.5** | **17.5** | **257.3** | **14.8** | **265.9** | **15.0** |
| P | 0.198 | | 0.126 | | **0.022** | | **0.032** | | **0.010** | | 0.115 | | **0.042** | | **0.018** | | **0.015** | |
| **HDL** <1.3mmol/l |  | | | | | | | | | | | | | | | | | |
| No (N=1081) | 255.0 | 20.1 | 323.0 | 16.3 | 321.3 | 16.1 | 308.9 | 15.4 | 317.8 | 16.5 | 295.6 | 16.3 | 279.4 | 15.0 | 260.3 | 14.2 | 269.0 | 15.4 |
| Yes (N=195) | 255.0 | 21.0 | 323.6 | 15.5 | 321.5 | 15.0 | 309.6 | 15.0 | 318.0 | 16.1 | 295.1 | 14.8 | 279.4 | 14.0 | 259.3 | 13.1 | 268.6 | 14.8 |
| P | 0.999 | | 0.592 | | 0.843 | | 0.595 | | 0.870 | | 0.671 | | 0.989 | | 0.351 | | 0.786 | |
