# Supplementary material for: Association between metabolic syndrome, macular structures and retinal vascular calibres in the Northern Finland Birth Cohort Eye Study
Source: Acta Ophthalmol. 2026 Apr 17;104(7):760–8. doi: 10.1111/aos.70131 (PMC13651912; doi:10.1111/aos.70131)
Supplement: Supplementary file 2 [file AOS-104-760-s002.docx]

Supplementary table 1. The macula’s thickness and the components of the MetS (N=2242) for men. Statistically significant values are highlighted.

| **MALES** | **Inner** | | | | | | | | | | **Outer** | | | | | | | |
| --- | --- | --- | --- | --- | --- | --- | --- | --- | --- | --- | --- | --- | --- | --- | --- | --- | --- | --- |
| **Macula** | Central subfield | | Nasal | | Superior | | Temporal | | Inferior | | Nasal | | Superior | | Temporal | | Inferior | |
|  | Mean (μm) | SD | Mean (μm) | SD | Mean (μm) | SD | Mean (μm) | SD | Mean (μm) | SD | Mean (μm) | SD | Mean (μm) | SD | Mean (μm) | SD | Mean (μm) | SD |
| **Overall** | 268.3 | 19.8 | 332.5 | 16.8 | 329.0 | 16.7 | 318.6 | 15.7 | 326.6 | 16.8 | 298.3 | 16.4 | 280.5 | 14.4 | 265.9 | 13.7 | 270.7 | 14.9 |
| **Waist circumference** >102cm |  | | | | | | | | | | | | | | | | | |
| No (N=659) | **269.4** | **20.0** | **333.5** | **16.9** | **329.7** | **16.5** | **319.3** | **15.7** | **327.4** | **16.9** | 299.0 | 16.4 | 281.0 | 14.2 | 266.3 | 13.8 | 271.3 | 15.2 |
| Yes (N=307) | **265.8** | **19.1** | **330.5** | **16.5** | **327.3** | **17.0** | **317.0** | **15.8** | **324.9** | **16.5** | 296.8 | 16.4 | 279.5 | 14.6 | 265.0 | 13.4 | 269.5 | 14.3 |
| P | **0.008** | | **0.010** | | **0.041** | | **0.033** | | **0.033** | | 0.053 | | 0.135 | | 0.165 | | 0.077 | |
| **Fasting plasma glucose** ≥6.1mmol/l |  | | | | | | | | | | | | | | | | | |
| No (N=779) | 268.3 | 19.5 | 333.0 | 16.8 | **329.5** | **16.7** | **319.1** | **15.6** | **327.2** | **16.7** | 298.8 | 16.6 | 280.9 | 14.6 | 266.2 | 13.7 | 271.1 | 14.9 |
| Yes (N=187) | 268.3 | 21.0 | 330.6 | 16.7 | **326.6** | **16.4** | **316.2** | **15.9** | **324.1** | **17.1** | 296.1 | 15.5 | 279.0 | 13.4 | 264.4 | 13.6 | 269.3 | 15.2 |
| P | 0.969 | | 0.085 | | **0.032** | | **0.025** | | **0.025** | | 0.036 | | 0.094 | | 0.097 | | 0.159 | |
| **Blood pressure** ≥130/85mmHg |  | | | | | | | | | | | | | | | | | |
| No (N=447) | 269.0 | 20.9 | **333.7** | **16.5** | 330.0 | 16.1 | **320.0** | **15.2** | **327.8** | **16.2** | 299.3 | 16.1 | 281.3 | 13.9 | **267.0** | **13.4** | 271.6 | 14.5 |
| Yes (N=519) | 267.6 | 18.8 | **331.5** | **17.0** | 328.0 | 17.1 | **317.4** | **16.1** | **325.5** | **17.3** | 297.4 | 16.7 | 280.0 | 14.8 | **264.9** | **13.8** | 269.9 | 15.3 |
| P | 0.261 | | **0.035** | | 0.060 | | **0.011** | | **0.036** | | 0.075 | | 0.144 | | **0.017** | | 0.075 | |
| **Triglycerides** ≥1.7mmol/l |  | | | | | | | | | | | | | | | | | |
| No (N=703) | 268.8 | 19.5 | **333.3** | **17.0** | 329.5 | 17.0 | 319.2 | 15.7 | 327.2 | 17.0 | **298.9** | **16.9** | 281.0 | 14.8 | 266.3 | 14.0 | 271.1 | 15.5 |
| Yes (N=263) | 266.8 | 20.5 | **330.6** | **16.1** | 327.5 | 15.8 | 317.0 | 15.7 | 325.0 | 16.1 | **296.6** | **14.9** | 279.4 | 13.3 | 264.7 | 12.8 | 269.6 | 13.3 |
| P | 0.183 | | **0.023** | | 0.091 | | 0.055 | | 0.071 | | **0.035** | | 0.116 | | 0.095 | | 0.115 | |
| **HDL** <1.0mmol/l |  | | | | | | | | | | | | | | | | | |
| No (N=892) | 268.3 | 19.8 | 332.6 | 16.8 | 329.0 | 16.7 | 318.7 | 15.8 | 326.7 | 16.8 | 298.4 | 16.6 | 280.6 | 14.6 | 265.8 | 13.8 | 270.8 | 15.2 |
| Yes (N=74) | 267.6 | 20.1 | 331.0 | 16.7 | 328.5 | 16.1 | 317.4 | 15.4 | 325.1 | 16.9 | 297.4 | 14.4 | 280.1 | 12.0 | 266.4 | 11.4 | 270.3 | 11.7 |
| P | 0.762 | | 0.416 | | 0.800 | | 0.502 | | 0.418 | | 0.573 | | 0.721 | | 0.667 | | 0.744 | |
